# Synthesis and Characterization of Rebondable Polyurethane Adhesives Relying on Thermo-Activated Transcarbamoylation

**DOI:** 10.3390/polym16192799

**Published:** 2024-10-03

**Authors:** Daniel Bautista-Anguís, Lisbeth Reiner, Florian Röper, Sebastian Maar, Markus Wolfahrt, Archim Wolfberger, Sandra Schlögl

**Affiliations:** Polymer Competence Center Leoben GmbH (PCCL), Sauraugasse 1, 8700 Leoben, Austria; daniel.bautista@pccl.at (D.B.-A.); lisbeth.reiner@tugraz.ac.at (L.R.); florian.roeper@ogi.at (F.R.); sebastian.maar@pccl.at (S.M.); markus.wolfahrt@pccl.at (M.W.);

**Keywords:** dynamic polymers, vitrimers, adhesives, thermo-activated transcarbamoylation, rebonding

## Abstract

Dynamic polymer networks combine the noteworthy (thermo)mechanical features of thermosets with the processability of thermoplastics. They rely on externally triggered bond exchange reactions, which induce topological rearrangements and, at a sufficiently high rate, a macroscopic reflow of the polymer network. Due to this controlled change in viscosity, dynamic polymers are repairable, malleable, and reprocessable. Herein, several dynamic polyurethane networks were synthetized as model compounds, which were able to undergo thermo-activated transcarbamoylation for the use in rebondable adhesives. Ethylenediamine-*N*,*N*,*N*′,*N*′-tetra-2-propanol (EDTP) was applied as a transcarbamoylation catalyst, which participates in the curing reaction across its four -OH groups and thus, is covalently attached within the polyurethane network. Both bond exchange rate and (thermo)mechanical properties of the dynamic networks were readily adjusted by the crosslink density and availability of -OH groups. In a last step, the most promising model compound was optimized to prepare an adhesive formulation more suitable for a real case application. Single-lap shear tests were carried out to evaluate the bond strength of this final formulation in adhesively bonded carbon fiber reinforced polymers (CFRP). Exploiting the dynamic nature of the adhesive layer, the debonded CFRP test specimens were rebonded at elevated temperature. The results clearly show that thermally triggered rebonding was feasible by recovering up to 79% of the original bond strength.

## 1. Introduction

Polymer-based adhesives have become indispensable in industry and our daily life. They are typically composed of thermosets or elastomers, whose covalent crosslinks ensure reliable bonding and high bond strength during the lifetime of the adhesive layer. However, the stable chemical bonds pose a challenge when it comes to the recycling or disassembling of composite structures [1,2]. Vast efforts have been dedicated to develop temporary adhesives, which lose their bond strength upon an external stimulus such as high energy irradiation [3], heat [4], electric [5], or magnetic fields [6]. Prominent de-bonding mechanisms rely on a controlled network degradation by cleavage reactions [7,8], incorporation of phase change materials, shift of the glass transition temperature by additional crosslinking reactions [9,10] and volumetric expansion [11,12]. Another strategy is the introduction of reversible binding motifs within the polymer network to reversibly switch on and off the bond strength as a response to two different external triggers [13]. However, whilst bonding and de-bonding strength are conveniently adjusted by network composition and/or appropriate surface functionalization, the clean and residue-free removal of the adhesive layer is still challenging.

Herein, to overcome this issue, the development of repairable and rebondable adhesive layers, whose residues are able to chemically bond to a new adhesive layer by exploiting the unique properties of dynamic covalent bonds, was studied. Dynamic covalent polymer networks have gained increased attention over the past years as they combine the advantages of thermosets and thermoplastics [14,15]. Polymer networks following a thermo-activated associative bond exchange mechanism retain their network connectivity at all temperatures until the degradation temperature is reached. However, in contrast to permanent crosslinked polymers, their viscosity is governed by the kinetics of the bond exchange reactions. At lower temperatures, the bond exchange progresses at a slow rate and the polymer behaves like a permanent crosslinked network. With rising temperature, the reaction rate is accelerated and facilitates topological rearrangements, which are observable in a macroscopic flow of the network. The rate of bond exchange reactions and the related macroscopic material flow follow an Arrhenian dependence with temperature. The temperature at which the gradual transition from a viscoelastic solid to a viscoelastic liquid occurs is termed topological freezing temperature (*T*_v_). Owing to their unique temperature-dependent viscoelastic properties, covalently crosslinked dynamic networks become repairable, reprocessable, recyclable, and weldable above their *T*_v_ [16,17,18].

The *T*_v_ is affected by various parameters including the kinetics of the exchange reaction, the number of dynamic bonds and functional groups, and the mobility of the polymer chains. As most systems rely on an appropriate catalyst for accelerating the bond exchange reactions, the *T*_v_ is also often governed by the type and availability of the catalyst [14].

The design and application of dynamic polymer networks is highly versatile, and numerous chemical reactions including olefin metathesis [19], boron ester exchanges [20,21,22], transamination of vinylogous urethanes [23,24,25,26], disulfide exchange [27,28,29,30], transesterification [31,32,33,34,35,36], transcarbamoylation [37] and vinylogous urethane transamination [26,38] have been reported over the past years.

In the present work, the catalyzed and thermo-activated transcarbamoylation reaction was employed to impart dynamic properties in polyurethane networks. Polyurethanes (PU) are well-established in the adhesive industry, and various approaches have already been reported to control the bond strength of this material class via dynamic bonds. Li and co-workers synthetized PU-based reversible adhesives, which were able to undergo boronic ester exchange reactions [39]. Although the networks were healable under mild conditions, they comprised a rather low bond strength. Advancing this concept, Jing et al. exploited nitrogen-coordinating cyclic boronic diester links to improve the temperature and hydrolytic stability of dynamic PU networks [40]. The group of Manas-Zloczower used a combination of transesterification and transcarbamoylation to heal PU networks [41]. By incorporating carbon nanotubes as functional fillers, they were able to rapidly heat up the polymer composite under microwave irradiation and heal macroscopic damages inserted into thin films.

In this study, a similar approach was followed and a PU network by addition reaction of 4,4′-methylene bis(phenylisocyanate) across polyethylene glycol was prepared. The degree of crosslinking and the thermo-mechanical properties were adjusted by adding glycerin as tri-functional alcohol. In contrast to previous studies, which typically applied organometallic compounds such as dibutyltin dilaurate, Zr(acac)_4_, Sn(Oct)_2_, or Ti(OiPr)_2_(acac)_2_ as transcarbamoylation catalyst, an amine base with free -OH groups was used [42]. Thus, the catalyst is directly incorporated into the network and immobilized, which prevents migration during the lifetime of the adhesive layer. Xie et al. [43] reported on the high efficiency of this base in catalyzing transcarbamoylation reactions and used it as a building block in synthetizing dynamic PU networks with triple shape memory properties.

In a systematic study, the temperature-dependent stress relaxation of the dynamic networks was determined as a function of their composition and tested the bonding performance of a promising formulation on carbon fiber-reinforced polymer (CFRP) substrates (with an epoxy-based resin as matrix material). Rebonding of the broken lap shear test specimen was then carried out above the network’s *T*_v_, and the recovered bond strength was characterized versus the time and temperature used in the repair process. 

## 2. Materials and Methods

### 2.1. Materials and Chemicals

Ethylenediamine-*N*,*N*,*N*′,*N*′-tetra-2-propanol (EDTP), polyethylene glycol with an average Mn of 400 (PEG400), 4,4′-methylenebis(phenylisocyanate) (MDI), glycerin, dibutyltin dilaurate (DBTDL), and anhydrous ethyl acetate were supplied by Merck (Darmstadt, Germany). Desmodur^®^ VL R 20 (pMDI: 2.9 functional groups) was provided by Covestro (Leverkusen, Germany). All chemicals were used as received.

### 2.2. Sample Preparation

For the preparation of the model compounds, EDTP, PEG, and glycerin were first separately heated at 40 °C for 45 min and then mixed together and diluted with ethyl acetate (0.5 mL per 1 g of PEG). The solution was stirred at room temperature for 30 min. Simultaneously, MDI was placed in a round neck flask, dissolved in ethyl acetate (1 g/mL), and stirred in an ice bath for 30 min. Once the EDTP-PEG-glycerin solution was added to the MDI solution, the formulation was stirred at 0 °C for 20 min. For the preparation of the various model compounds, the molar ration between EDTP and PEG was varied together with the glycerin content. The composition of the formulations under investigation is provided in Table 1. Moreover, a reference formulation using DBTDL as conventional catalyst instead of EDTP was prepared. Here, the catalyst was dissolved in the alcohol solution prior to the mixing with the MDI solution. Model compounds were casted in aluminum pans cured in an oven at 60 °C for 90 min, followed by a treatment at 120 °C for 90 min. Any remaining volatile species was removed in the last curing step involving a treatment at 60 °C for 90 min in a vacuum oven.

A final formulation (S5) was developed combining EDTP (1 mmol), PEG (10 mmol), and pMDI (8.28 mmol) following a stoichiometric OH:NCO ratio of 1:1. Transition from MDI to pMDI was done with the purpose of using a commercially available polymeric variation of MDI, already provided in a liquid state and close to a real application, which can avoid the use of high amount of solvents and can be stored at standard conditions and not at freezing temperatures as typical for the pure MDI. Single-lap shear samples were prepared using S5 as adhesive and CFRP coupons (matrix material was an epoxy-based resin), previously cleaned with isopropanol, as substrates. In order to ensure a constant bonding area of 25 mm × 12.5 mm, a custom-made compactor was used (see Figure S1 in the supporting information). The above-described curing steps for S0–S4 were followed as well for the preparation of single-lap shear tests using S5. 

### 2.3. Fourier-Transform Infrared (FT-IR) Spectroscopy

FT-IR measurements were done on a Bruker Vertex 70 (Brucker Optics GmbH & Co. KG, Ettlingen, Germany) in attenuated total reflectance (ATR) mode. A total of 16 scans with a resolution of 4 cm^−1^ were accumulated. Peak locations as well as absorption peak intensities were determined by Origin (Version 9.0) from OriginLab Corporation.

### 2.4. Thermogravimetric Analysis (TGA)

TGA measurements were conducted using a TGA/DSC 1 and a TGA/DSC 3 thermogravimetric analyzers (Mettler Toledo, OH, USA). Tests were carried out from 25 °C to 900 °C using a heating ramp of 10 K/min under a N_2_ atmosphere (50 mL/min flow rate). Samples of 10–20 mg were prepared and tested in Al_2_O_3_ crucibles with a volume of 70 μL. Each sample was tested once by TGA. STARe Evaluation Software Version 16.10 from Mettler Toledo and Origin (Version 9.0) from OriginLab Corporation were used to calculate the reported results.

### 2.5. Differential Scanning Calorimetry (DSC)

DSC experiments were performed on a DSC 4000 instrument (Perkin Elmer, MA, USA). Aluminum crucibles of 50 μL volume, with punched lids to avoid overpressure events, were employed. Solid samples of approximately 7.5 mg were weighted for the model compounds (S0–S4). The obtained heat flow was normalized based on the sample weight. Under a N_2_ atmosphere (50 mL/min flow rate), samples were measured from −50 °C to 200 °C with a heating rate of 10 K/min, and two heating runs were conducted. One test was performed for each sample, and the second heating ramps were reported. Data evaluation was done using the softwares Pyris (Version 13.2.1.0007) from Perkin Elmer and Origin (Version 9.0) from OriginLab Corporation.

### 2.6. Stress-Relaxation Tests

A Physica MCR 501 Rheometer (Anton Paar, Graz, Austria) with a parallel-plate geometry setup was used for measuring the characteristic stress relaxation of the samples. Circular specimens with a 10 mm diameter and similar thickness were prepared and tested at different temperatures under a constant normal force of 15–20 N with a deformation of 3%. Measuring points were recorded every 2 s, and tests were stopped after the normalized relaxation module (G/G_0_) reached the value of 1/e. Single tests were done for each temperature and sample. The obtained data was processed and analyzed with the RheoCompass (Version 1.3.0) and Origin (Version 9.0) from OriginLab Corporation. 

### 2.7. Healing Tests

In order to study the reprocessability of the model compounds, an empirical test was performed in a first step. For this, cured samples were cut into small pieces and brought into contact by pressing them between two aluminum plates, which were squeezed by standard screw clamps. Afterwards, the prepared setup was placed in a convection oven at different times and temperatures to analyze the behavior of the samples.

### 2.8. Single-Lap Shear Tests

Single-lap shear tests were performed on a universal tensile-compression testing machine (ZwickRoell GmbH & Co. KG, Ulm, Germany) with a 10 kN load cell. The clamping length was 112.5 mm (clamping jaws: pneumatic 2.5 kN) while the applied testing speed was 2 mm/min. Three specimens were tested after bonding and rebonding. Softwares testXpert III (V1.9) from ZwickRoell and Origin (Version 9.0) from OriginLab Corporation were used for the evaluation of the data.

## 3. Results and Discussion

Inspired by the work of Xie et al. [43], dynamic polyurethane (PU) networks by mixing MDI and the polymeric pMDI with varying concentrations of PEG400 and EDTP were prepared. It was reported that the tertiary amine groups in EDTP are highly efficient in catalyzing transcarbamoylation-based exchange reactions at elevated temperatures. In the presence of the catalyst and free -OH groups, the mechanism of this type of bond exchange is mainly associative in nature. Associative dynamic networks are characterized by a constant crosslink density as in the exchange mechanism a new covalent bond is formed before a bond is broken [44,45].

**Figure 1:**
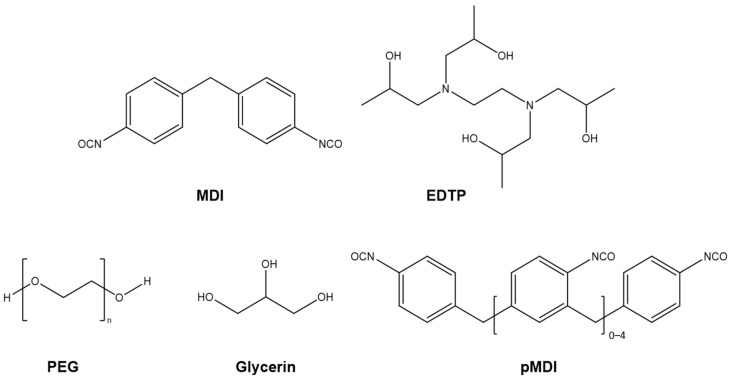
Chemical structures of the components used in the formulations.

The formulations described in the work of Xie et al. were used as a basis and modified in order to obtain networks with higher crosslink degrees, replacing part of PEG400 by glycerin as multi-functional and low molecular weight crosslinker in selected formulations (S2 and S3). S4 was formulated leaving unreacted and free -OH groups to study their effect in the bond exchange mechanism. The chemical structures of the components are provided in Figure 1. To evaluate the efficiency of the immobilized EDTP, a network using DBTL as a classical transcarbamoylation catalyst was further prepared (S0).

**Figure 2:**
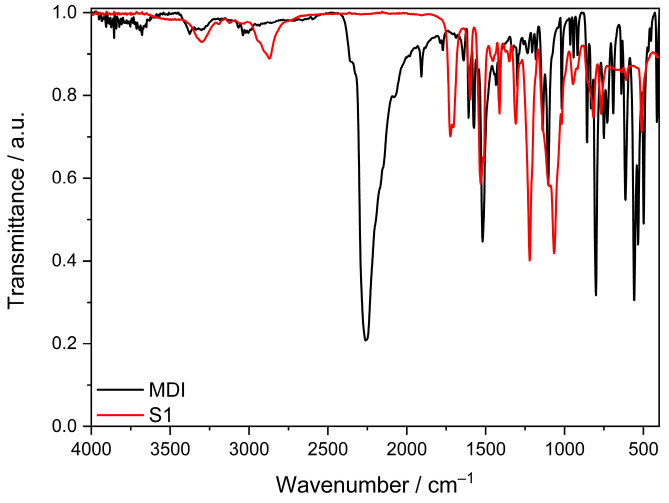
FT-IR spectra of pure MDI (black) and the thermally cured model compound S1 (red).

The progress of the curing reaction was studied by FT-IR spectroscopy, and as an example, Figure 2 shows the FT-IR data of the S1 formulation after carrying out the thermal curing steps described in the experimental section in comparison to pure MDI. Complete curing of the model network is confirmed by the disappearance of the characteristic NCO absorption peak at 2275 cm^−1^, whilst the peak arising at 1726 cm^−1^ is related to the formed urethane bond. The FT-IR spectra of S2, S3, and S4 can be found in the supporting information (Figure S2).

**Figure 3:**
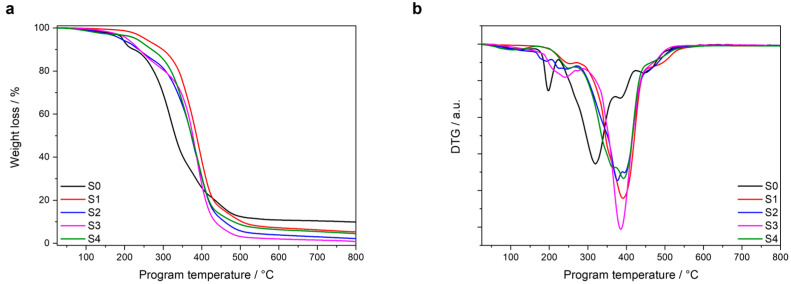
(**a**) TGA and (**b**) DTG curves of thermally cured model compounds.

To study the thermal stability of the polyurethane networks under investigation, thermogravimetric analysis (TGA) was carried out. For the compounds prepared with MDI (S0–S4), the normalized weight loss together with the first derivative (DTG) of the TGA curves are depicted as a function of the programmed temperature in Figure 3a,b. Two reference temperatures, *T*_ons1_ and *T*_ons2_, were defined and calculated as the temperature values in which each formulation loses 1% and 5% of their initial weight, respectively. The onset values are shown in Table 2.

S2 and S3, both containing EDTP and glycerin in their network, showed a lower thermal stability than S1 and S4 containing only EDTP but without glycerin. Sample 1, formulated with a stoichiometric OH:NCO ratio, presented the highest *T*_ons1_ (184 °C), and *T*_ons2_ (253 °C) being the most thermally stable formulation. S1 presented a higher thermal stability than S0 as a consequence of the higher crosslink density obtained when exchanging the classic tin catalyst (S0) with the amine-based monomer (providing four additional -OH groups for crosslinking) in S1. On the other hand, it can be assumed that using the same OH:NCO ratio (S1 and S2), a higher amount of MDI (S1) results in higher thermal stability. The presence of more EDTP in the formulation S3 increases its crosslinking density and therefore, explains its higher thermal stability with respect to formulation S2. Despite the fact that S1 and S4 have similar compositions, a difference in their thermal stability was observed. The lower thermal stability of S4 is related to the excess of -OH groups in the networks OH:NCO ratio of 1:0.95), indicating that a low number of unreacted -OH groups might have a negative impact on the thermal stability.

**Figure 4:**
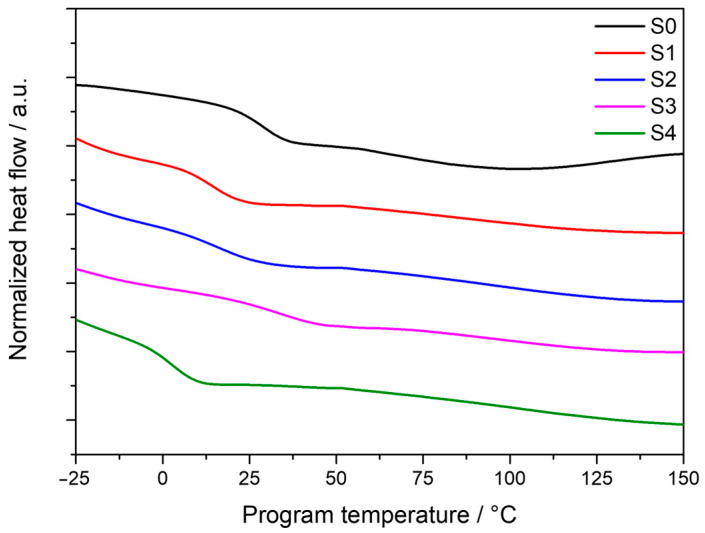
DSC curves of cured model compounds.

Glass transition temperatures (*Tg*) of the formulations were characterized by differential scanning calorimetry (DSC) measurements. *Tg* onset and *Tg* inflection point were calculated based on the tangent method. The obtained curves are shown in Figure 4. S3 containing glycerin and a non-stoichiometric OH:NCO ratio showed the highest *Tg* onset and *Tg* inflection point values, 20.4 °C and 33.3 °C, respectively. Those values were similar to ones obtained for the reference sample (S0), formulated without EDTP and glycerin. Samples with EDTP and formulated following a stoichiometric OH:NCO ratio, S1 and S2, showed similar *Tg* onset and *Tg* inflection point values. On the other hand, the lowest *Tg* values were measured for Sample 4, with a *Tg* onset of −4.1 °C and a *Tg* inflection point of 1.8 °C. A summary of the *Tg* values of each formulation is provided in Table 3. The introduction of the amine-based monomer leads to greater heterogeneity of the sample, thus explaining that S1 has a lower glass transition temperature than S0. The effect of using a similar stoichiometric OH:NCO ratio is clearly noticeable for S1 and S2, where both *Tg* values are identical. Increasing the concentration of MDI and EDTP results in a higher glass transition temperature as consequence of a minor chain mobility. This can be confirmed by comparing the glass transition temperatures of S2 and S3, where S3, with a higher concentration of MDI and EDTP, showed higher *Tg* values. Similar behavior was found for S1 and S4, where a decrease of the MDI concentration in S4 resulted in lower *Tg* values with respect to S1.

The stress relaxation modulus of the model compounds was measured at different temperatures to analyze the behavior of the dynamic bond exchange reactions. The time at which every network reaches the relaxation modulus value of 1/e, i.e., the 37% of the initial deformation, indicates the rate of the exchange reactions to complete the topological rearrangements at the measured temperature. This value is defined as the characteristic relaxation time of a covalent adaptable network system at a defined temperature [46].

**Figure 5:**
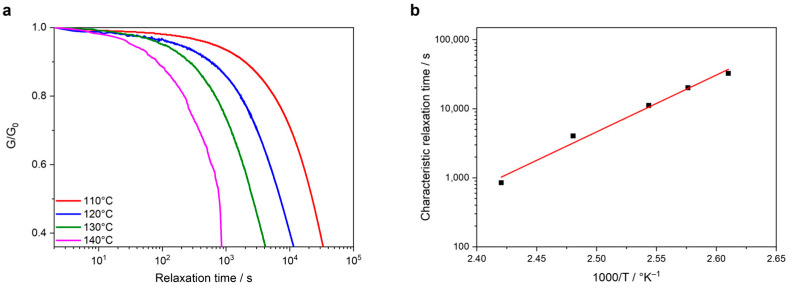
(**a**) Stress-relaxation curves at different temperatures and (**b**) linear correlation relaxation time-temperature of S1.

The most thermally stable sample, Sample 1, was firstly measured at different temperatures, obtaining a fastest relaxation time of around 17 min at 140 °C (Figure 5a). In order to observe the temperature dependence of the characteristic relaxation times, the characteristic relaxation times were plotted in a logarithmic scale against the inverse values of the measured temperatures, obtaining an Arrhenius behavior in which a clear correlation between the relaxation time and the temperature can be observed; the increase of the temperature induces a faster dynamic bond exchange (Figure 5b). A similar relaxation time-temperature behavior was noted for the other model compounds tested at different temperatures (Figures S3 and S4 in the supporting information).

Based on the Arrhenius relation (τ_(T)_ = τ_0_ exp (*E_a_*/RT)), the activation energy (*E*_a_) of the studied systems can be calculated as the slope of the linear regression. Table 4 summarized the activation energy of each system as well as the coefficient of determination (R^2^). S0 shows the highest activation energy. A higher activation energy is translated into a higher effort to reach a complete relaxation. That means that the samples prepared using EDTP as a covalently bonded transcarbamoylation catalyst are more easily reprocessable compared to S0, formulated with a conventional catalyst, meaning that the tertiary amine groups are highly efficient in catalyzing the exchange reactions.

It can also be proposed that the presence of glycerin in S2 and therefore, the presence of a higher variety of molecules with -OH groups, enables a faster transcarbamoylation than in S1 even though both systems are formulated using a stoichiometric OH:NCO ratio. Moreover, the unreacted -OH groups of S3 explain why the energy of activation of S3 is lower than S2 when both networks are formulated with the same monomers. The already available -OH groups can take part in the bond exchange reactions, speeding up the relaxation process. For the same reason, the presence of unreacted -OH groups in S4 explains the lower activation energy obtained for S4 in comparison to S1, even though both formulations contain the same components. The lower *E*_a_ of S4 with respect to S2 is also attributed to the free and unreacted -OH groups in S4.

**Figure 6:**
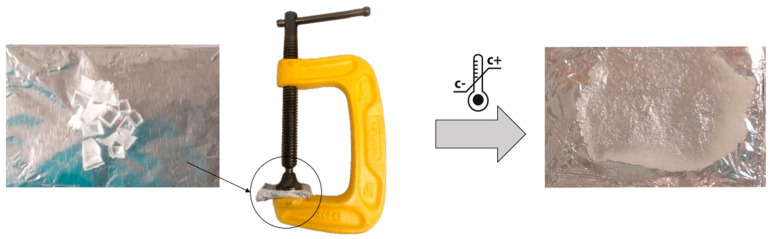
Schematic representation of the reprocessing tests.

Based on the results obtained in the stress-relaxation measurements, healing tests were performed to visually investigate the reprocessability of the studied samples as described in Figure 6. Cured samples from S1–S4 were manually cut in small pieces of approximately 0.5 cm and were then placed between two aluminum plates. The aluminum plates were covered with aluminum foil in order to avoid any adherence of the material on the surfaces of the aluminum plates. Afterwards, the aluminum plates were pressed together using a mechanical clamping device and heated at different temperatures (100–130 °C) for 30 min. Promising results were obtained when heating the samples for 30 min at 130 °C. Here it was probed that the cut pieces can be brought together following specific reprocessing parameters, obtaining one homogenous and optically transparent reprocessed sample.

Once the reprocessability and thermal properties of the model compounds using MDI as isocyanate component were extensibility studied, a final formulation (S5) closer to a real case system was developed using a commercially available polymeric MDI (Desmodur^®^ VL R 20), polyethylene glycol (PEG) and also EDTP as a covalently bonded catalyst. Since S1 was probed to be the most thermally stable formulation and it also showed an optimal relaxation process, S5 was formulated without glycerin and using a stoichiometric OH:NCO ratio.

Samples were prepared in a similar way as the model compounds. However, a lower amount of solvent was needed since the polymeric MDI was supplied in a liquid state, allowing a good mixing with the other components. Samples were poured and cured in an aluminum mold as described in the experimental section. 

**Figure 7:**
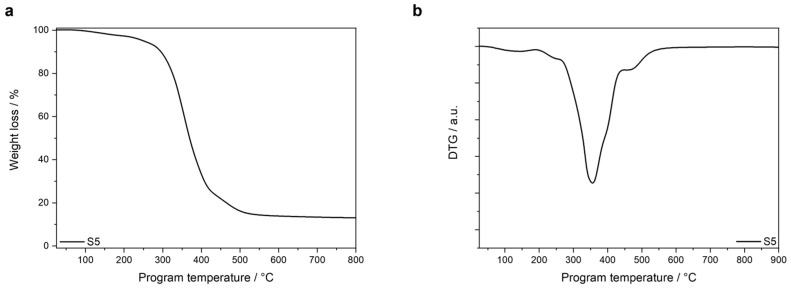
(**a**) TGA curves and (**b**) DTG curves of cured S5.

The final formulation was also thermally characterized by TGA to analyze the stability. *T*_ons1_ and *T*_ons2_ were measured at 126 °C and 251 °C, respectively, being both values within the same temperature range as *T*_ons1_ and *T*_ons2_ of the model compounds. The obtained TGA and DTG curves are showed in Figure 7.

**Figure 8:**
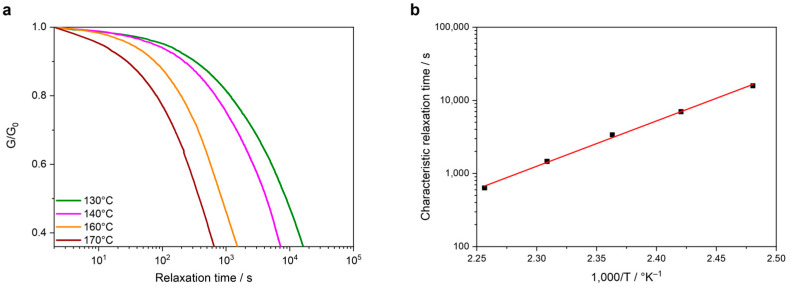
(**a**) Stress-relaxation curves at different temperatures and (**b**) linear correlation relaxation time-temperature of S5.

As for the model compounds, the stress-relaxation behavior at different temperatures of S5 was measured. A fast-relaxation behavior was observed at temperatures between 130 °C and 170 °C (Figure 8a). A coefficient of determination of 0.99 was determined, stating the strong linear correlation between the relaxation time and the test temperature. Moreover, using the aforementioned Arrhenius relation, an activation energy of 119 kJ/mol was calculated (Figure 8b).

To study the reprocessability of S5, single-lap shear test specimens were tested prior to and after reprocessing. The formulation was carefully done by manually applying the same volume on a 25 mm × 12.5 mm bonding surface between two CFRP substrates using a custom-made tool provided by CEST Kompetenzzentrum für elektrochemische Oberflächentechnologie GmbH (Wiener Neustadt, Austria) (Figure S1) that, when closed, provides a constant and homogeneous pressure (Figure 8). The excess of resin was squeezed out after closing the tool and was partially removed before testing.

**Figure 9:**
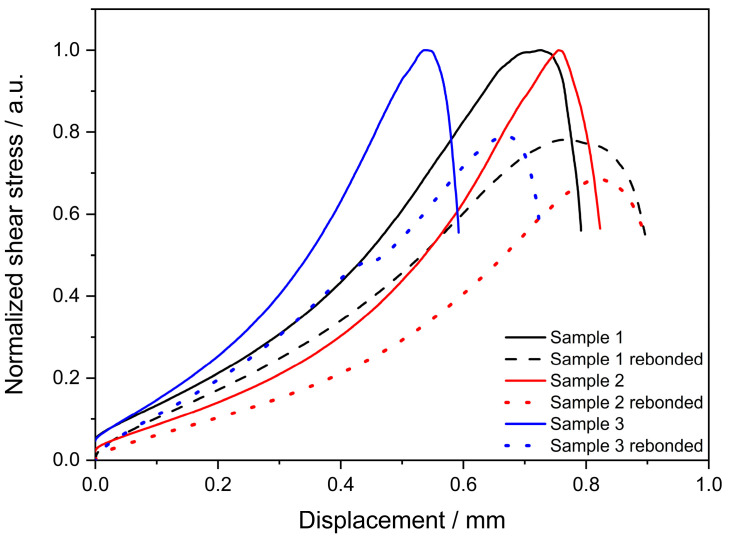
Normalized lap shear test curves of S5 samples after bonding and rebonding.

The curing process was carried out in a convection oven, first at 60 °C for 90 min and subsequently, at 120 °C for 90 min. A final post-curing step was done at 60 °C under vacuum conditions. After the first batch of samples was tested, the de-bonded parts were brought together again using the tool, ensuring the same alignment and position as in the previous sample preparation. Rebonding was carried out at 150 °C for 5 h, and the samples were tested again. Promising recovery values (75 ± 5%) were calculated when comparing the results prior (2 ± 0.5 MPa) to and after (1.5 ± 0.3 MPa) reprocessing. Figure 9 shows the normalized single-lap shear values for three samples measured before and after reprocessing. A re-workability of the formulated systems is here confirmed by a recovery of up to 79% of their initial adhesive properties. Single test curves can be found in the supporting information (Figure S5). These findings are of great value as a basis for future adhesive development that can be reused or recycled in different industrial applications, from structural, such as joining of composite parts, to aesthetic applications, such as cladding or publicity panels. 

## 4. Conclusions

The present study successfully developed six different dynamic polyurethane formulations (S0–S5) with varying compositions and evaluated their reworkability. The work confirmed that the use of EDTP as a covalently bonded catalyst was highly effective at catalyzing the transcarbamoylation bond exchange reactions. Firstly, model compounds (S1–S4) were prepared using MDI, glycerin, PEG, and EDTP and compared with a model compound (S0) formulated with a standard tin catalyst. Model compounds were thermally characterized, being the formulations without glycerin (S1 and S4) the most thermally stable. S1 formulated with a stoichiometric OH:NCO ratio showed the highest thermal stability. The use of glycerin and a non-stoichiometric OH:NCO ratio resulted in higher *Tg* values. EDTP-based networks displayed a higher reprocessability than networks with a conventional catalyst due to the efficient catalyst effect of the tertiary amine. The higher amount of unreacted -OH groups explained the lowest activation energy, measured for S3. The reprocessability of model compounds was also probed by a home-made test, where a broken sample was heated up under pressure obtaining a compact and homogenous layer again. Following the components ratio of the most thermally stable model compound (S1), S5 was formulated using a commercially available polymeric MDI instead of MDI. The thermal stability at high temperatures was measured by TGA and the bond exchange reaction by stress relaxation test. Moreover, the reworkability of S5 was confirmed by single-lap shear tests, prepared and reprocessed using a custom-made tool, observing that the adhesive properties of the system after reprocessing were retained up to 78%.

Despite the advantages of polyurethane adhesive systems, some limitations can affect their use in specific industrial applications. One of the most critical points is the sensitivity to moisture during processing, affecting the final properties, which could lead to a weak adhesion. Moreover, these adhesive systems present lower resistance to high temperatures than epoxy-based adhesives used in similar applications, thus limiting their use in applications where high temperatures are required. In general, polyurethane adhesives suffer from a lower resistance to high energy radiation and can degrade after long-time exposure to ultraviolet radiation. This represents a further limitation when employing PU adhesives in outdoor applications.

The presented PU formulations were developed with the main aim to prove and test their reprocessability by the exploiting EDTP as a covalently bonded catalyst, to underscore their potential for future industrial applications. Whilst the obtained results can definitely be used as a basis in the development of reusable adhesives, a further optimization of the network structure is required to provide materials with a higher bond strength.

## Figures and Tables

**Table 1:** Composition of model compounds.

Formulation ID	MDI mmol	PEG mmol	Glycerine mmol	EDTP mmol	DBTDL wt. %	OH:NCO Ratio
S0	1	1	-	-	1.5	-
S1	7	5	-	1	-	1:1
S2	5.5	3	0.5	1	-	1:1
S3	7.8	3	0.5	3	-	1:0.8
S4	6.6	5	-	1	-	1:0.95

**Table 2:** TGA onset values of thermally cured model compounds.

Formulation	*T*_ons1_/°C	*T*_ons2_/°C
S0	114	195
S1	184	253
S2	105	190
S3	128	206
S4	92	230

**Table 3:** *Tg* data (obtained from DSC measurements) of cured model compounds.

Formulation	*Tg* Onset/°C	*Tg* Inflection Point/°C
S0	20	29
S1	6	15
S2	5	15
S3	20	33
S4	−4	2

**Table 4:** Activation energy and coefficient of determination of model compounds.

Formulation	*E*_a_	R^2^
S0	185 kJ/mol	0.99
S1	158 kJ/mol	0.99
S2	150 kJ/mol	0.98
S3	104 kJ/mol	0.98
S4	138 kJ/mol	0.99

## Data Availability

The original contributions presented in the study are included in the article/Supplementary Materials. Further inquiries can be directed to the corresponding author/s.

## Supplementary Materials

The following supporting information can be downloaded at: https://www.mdpi.com/article/10.3390/polym16192799/s1, Figure S1: Custom-made compactor used for the preparation and reprocessing of single-lap shear samples. Provided by CEST Kompetenzzentrum für elektrochemische Oberflächentechnologie GmbH; Figure S2: FT-IR spectra of MDI and the thermally cured model compounds S2, S3 and S4; Figure S3: Stress-relaxation curves at different temperatures for S0 (a), S2 (b), S3 (c) and S4 (d); Figure S4: Linear correlation relaxation time-temperature for S0 (a), S2 (b), S3 (c) and S4 (d); Figure S5: Lap shear tests curves of S5 samples after bonding and rebonding.
