# Supplementary material for: Synthesis and Characterization of Rebondable Polyurethane Adhesives Relying on Thermo-Activated Transcarbamoylation
Source: Polymers (Basel). 2024 Oct 3;16(19):2799. doi: 10.3390/polym16192799 (PMC11478668; doi:10.3390/polym16192799)
Supplement: Supplementary file 1 [file polymers-16-02799-s001.zip › polymers-3156011-supplementary.pdf]

Supporting Information

Synthesis and characterization of rebondable polyurethane adhesives relying on thermo-activated transcarbamoylation

Daniel Bautista-Anguís[1], Lisbeth Reiner[1], Florian Röper[1], Sebastian Maar[1], Markus Wolfahrt[1], Archim Wolfberger[1], Sandra Schlögl[1*]

[1]Polymer Competence Center Leoben GmbH (PCCL), Sauraugasse 1, 8700 Leoben, Austria

E-mail: sandra.schloegl@pccl.at

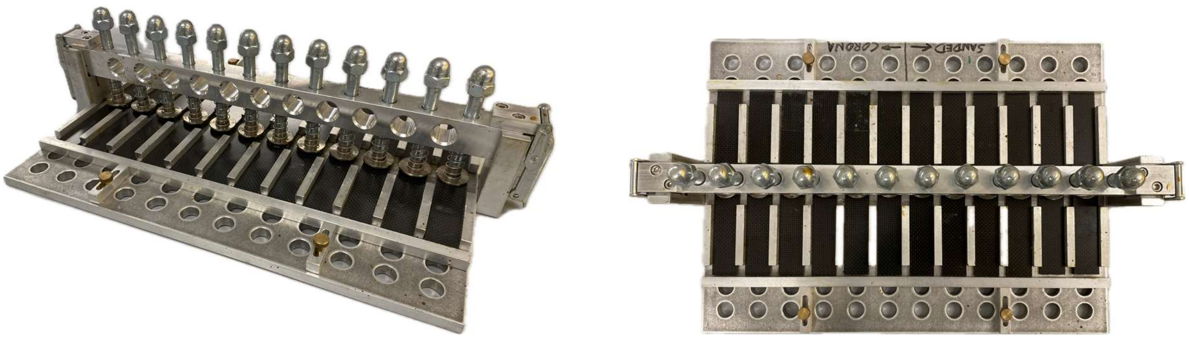

Figure S1 - Custom-made compactor used for the preparation and reprocessing of single lap shear samples. Provided by CEST Kompetenzzentrum für elektrochemische Oberflächentechnologie GmbH

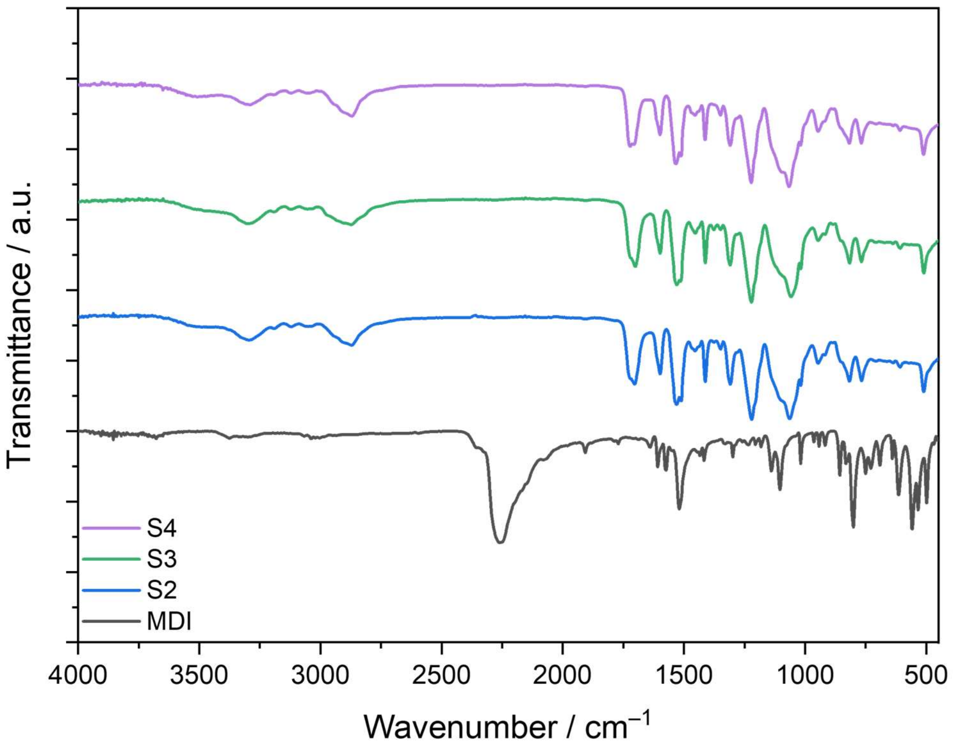

Figure S2 - FT-IR spectra of MDI and the thermally cured model compounds S2, S3 and S4

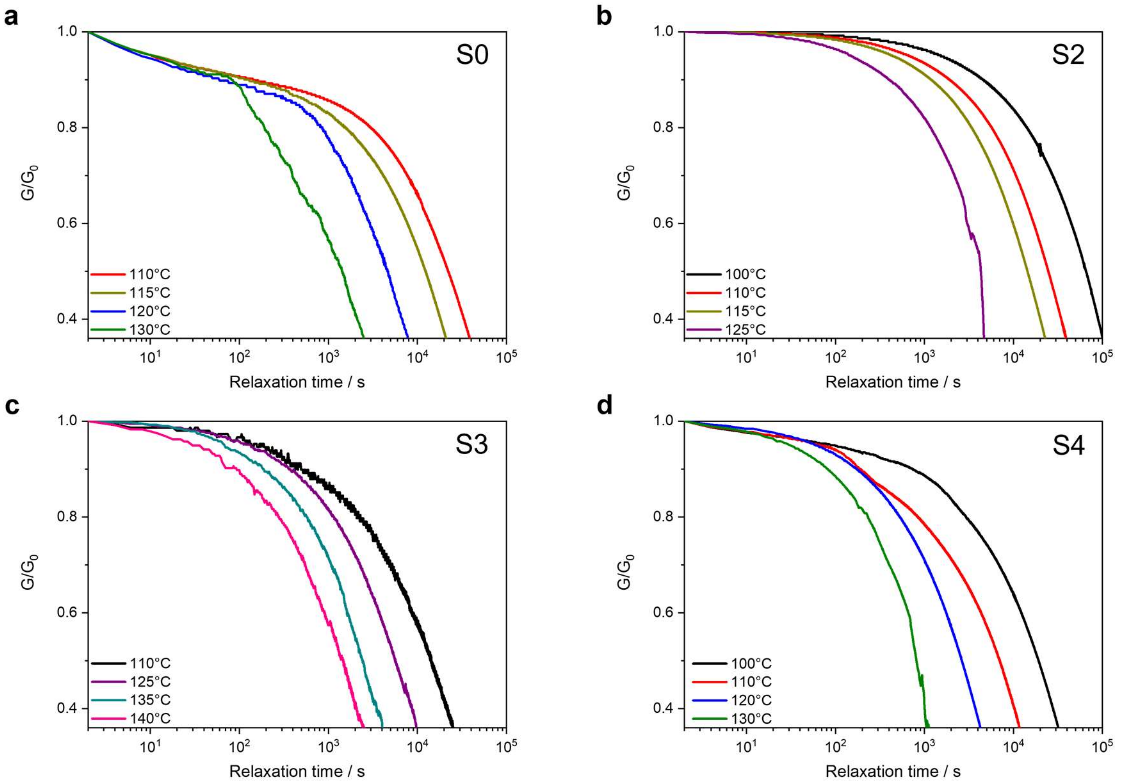

Figure S3 - Stress-relaxation curves at different temperatures for S0 (a), S2 (b), S3 (c) and S4 (d)

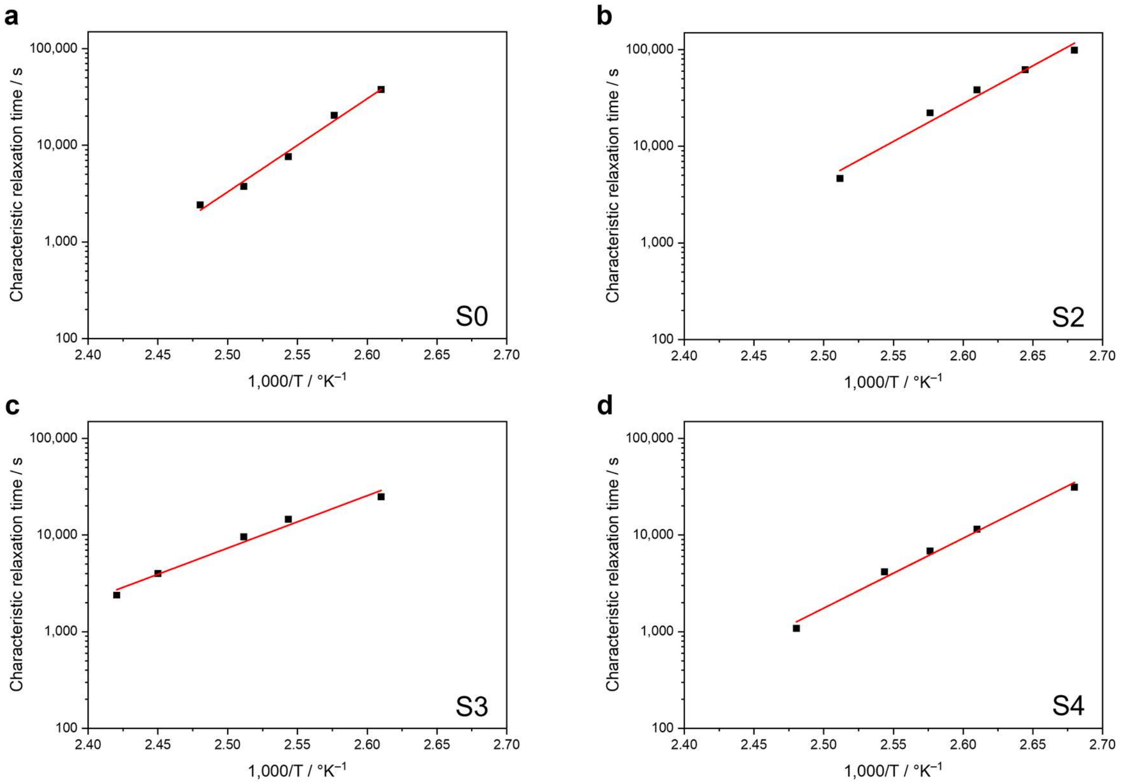

Figure S4 - Linear correlation relaxation time-temperature for S0 (a), S2 (b), S3 (c) and S4 (d)

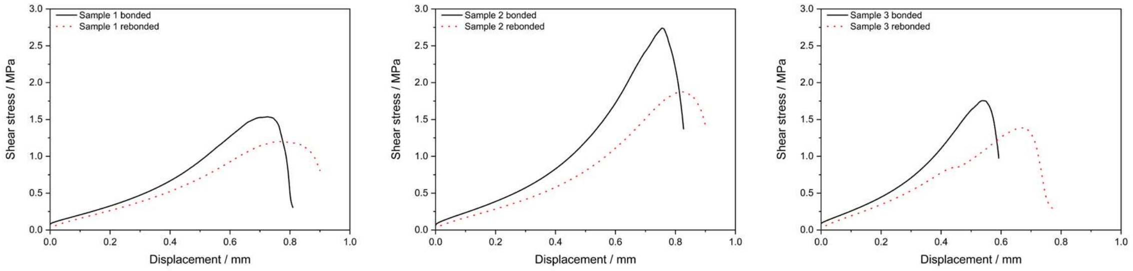

Figure S5 - Lap shear tests curves of S5 samples after bonding and rebonding
